# Resistance mechanisms to osimertinib in *EGFR*-mutated non-small cell lung cancer

**DOI:** 10.1038/s41416-019-0573-8

**Published:** 2019-09-30

**Authors:** Alessandro Leonetti, Sugandhi Sharma, Roberta Minari, Paola Perego, Elisa Giovannetti, Marcello Tiseo

**Affiliations:** 1 Medical Oncology Unit, University Hospital of Parma, 43126 Parma, Italy; 2 Department of Medical Oncology, Amsterdam University Medical Center, VU University, 1081 HV Amsterdam, Netherlands; 3 Molecular Pharmacology Unit, Department of Applied Research and Technological Development, Fondazione IRCCS Istituto Nazionale dei Tumori, 20133 Milan, Italy; 4 Cancer Pharmacology Lab, AIRC Start-Up Unit, Fondazione Pisana per la Scienza, 56017 Pisa, Italy; 5 Department of Medicine and Surgery, University of Parma, Parma, Italy

**Keywords:** Non-small-cell lung cancer, Cancer therapeutic resistance

## Abstract

Osimertinib is an irreversible, third-generation epidermal growth factor receptor (EGFR) tyrosine kinase inhibitor that is highly selective for *EGFR-*activating mutations as well as the *EGFR* T790M mutation in patients with advanced non-small cell lung cancer (NSCLC) with *EGFR* oncogene addiction. Despite the documented efficacy of osimertinib in first- and second-line settings, patients inevitably develop resistance, with no further clear-cut therapeutic options to date other than chemotherapy and locally ablative therapy for selected individuals. On account of the high degree of tumour heterogeneity and adaptive cellular signalling pathways in NSCLC, the acquired osimertinib resistance is highly heterogeneous, encompassing EGFR*-*dependent as well as EGFR-independent mechanisms. Furthermore, data from repeat plasma genotyping analyses have highlighted differences in the frequency and preponderance of resistance mechanisms when osimertinib is administered in a front-line versus second-line setting, underlying the discrepancies in selection pressure and clonal evolution. This review summarises the molecular mechanisms of resistance to osimertinib in patients with advanced *EGFR-*mutated NSCLC, including *MET/HER2* amplification, activation of the RAS–mitogen-activated protein kinase (MAPK) or RAS–phosphatidylinositol 3-kinase (PI3K) pathways, novel fusion events and histological/phenotypic transformation, as well as discussing the current evidence regarding potential new approaches to counteract osimertinib resistance.

## Background

The identification of activating mutations in the gene encoding the epidermal growth factor receptor (EGFR) tyrosine kinase in patients with non-small cell lung cancer (NSCLC) and the subsequent development of targeted therapy with small-molecule EGFR tyrosine kinase inhibitors (TKIs) has dramatically revolutionised the treatment landscape of these tumours. Somatic, activating mutations in the tyrosine kinase domain of *EGFR* are present in about 15% of Caucasian and nearly 50% of Asian patients with advanced NSCLC.^1,2^ Almost 90% of these mutations consist of deletions in exon 19 or L858R point mutations within exon 21. These genetic changes act as oncogenic drivers leading to ligand-independent activation of EGFR downstream signalling, thus promoting cell proliferation, survival and migration.

Several large-scale Phase 3 clinical trials have consistently demonstrated the superior efficacy of first-generation (gefitinib and erlotinib) and second-generation (afatinib) TKIs in comparison with standard first-line platinum-based chemotherapy for the treatment of patients with advanced NSCLC with activating *EGFR* mutations.^3^ Unfortunately, despite a remarkably high (60–70%) objective response rate (ORR) to such treatments, the majority of patients develop resistance, with average progression-free survival (PFS) ranging from 9 to 15 months.^3^ The most common resistance mechanism results from the development of the so-called ‘gatekeeper’ T790M mutation in *EGFR* exon 20, which sterically hinders the binding of first- and second-generation TKIs to the ATP-binding site of EGFR.^4^ However, this limitation has been overcome by the introduction of third-generation TKIs, particularly osimertinib. Osimertinib (Tagrisso™, [AZD9291] AstraZeneca, Cambridge, UK) is an orally administered EGFR-TKI that selectively targets activating *EGFR* mutations, as well as the T790M-resistance mutation, through the formation of a covalent bond to the C797 residue in the ATP-binding site of mutant EGFR. Compared with first- and second-generation EGFR-TKIs, osimertinib demonstrated a superior activity against T790M mutants in vitro, with minimal off-target effects resulting, when tested in patients, in fewer adverse events usually associated with the blockade of wild-type (wt) EGFR.^5^ Currently, osimertinib is the only third-generation EGFR-TKI approved by major regulatory agencies for treatment of T790M-positive patients who have progressed on first- or second-generation EGFR-TKIs. Osimertinib was also approved in 2018 as first-line therapy for advanced *EGFR*-mutated NSCLC, regardless of T790M mutation status.^6^ However, despite the robust clinical activity exerted by osimertinib, patients inevitably develop secondary resistance to this treatment, which poses a significant challenge due to the paucity of post-osimertinib pharmacological options available to date. The aim of this review is to provide a comprehensive overview of resistance mechanisms to osimertinib and to take advantage of the available knowledge to build future strategies and gain insights to overcome resistance to this agent.

## Osimertinib therapy

### Osimertinib treatment after failure of previous EGFR-TKI therapy

The clinical efficacy of osimertinib for the treatment of patients with advanced *EGFR*-mutated NSCLC who had experienced disease progression during prior therapy with EGFR-TKIs was first documented in the international Phase 1/2 AURA trial.^7^ In this study, osimertinib achieved an ORR of 61% (95% confidence interval [CI]: 52–70) among patients with centrally confirmed T790M mutations (*n* = 127). In the same population, median PFS was 9.6 months (95% CI: 8.3 to not reached) and the recommended dose for future trials was set at 80 mg/day.^7^ The subsequent AURA Phase 2 extension study and the open-label Phase 2 AURA2 trial confirmed the safety and efficacy of osimertinib as a post-EGFR-TKI treatment in patients with *EGFR*-mutated NSCLC with the T790M mutation.^8,9^

Osimertinib was further investigated in the randomised international Phase 3 AURA3 trial, which compared this compound with platinum–pemetrexed combination chemotherapy in EGFR T790M-positive NSCLC patients following progression during prior EGFR-TKI treatment.^10^ The median PFS for patients treated with osimertinib was more than twice that of those treated with platinum-based chemotherapy (10.1 versus 4.4 months, hazard ratio [HR]: 0.30; 95% CI: 0.23–0.41, *P* < 0.001). In addition, ORR for osimertinib was 71% compared with 31% for platinum-based chemotherapy (*P* < 0.001). Remarkably, the treatment with osimertinib was also well tolerated, with a lower incidence of grade 3 adverse effects than the chemotherapy arm (23% versus 47%).^10^ The long-term follow-up data from a pre-planned pooled analysis of AURA and AURA2 trials confirmed the efficacy of osimertinib in T790M-positive EGFR-TKI pre-treated patients.^11^ The median overall survival (OS) was 26.8 months (95% CI: 24.0–29.1 months) and the 12-month survival rate was 80%.^11^ These striking results led to the Food and Drug Administration (FDA) approval of osimertinib for the treatment of patients with metastatic *EGFR*-mutated NSCLC with an acquired T790M mutation after progression on previous EGFR-TKI therapy.

### Osimertinib as first-line therapy

Following the impressive clinical results shown by osimertinib as a salvage therapy in the presence of the T790M secondary mutation, first-line treatment with osimertinib in an attempt to avoid this acquired resistance was investigated.^6,12^ The FLAURA Phase 3 trial directly compared the efficacy and safety of osimertinib with standard first-generation EGFR-TKIs (gefitinib or erlotinib) in 556 previously untreated patients with advanced, *EGFR* mutation-positive NSCLC.^6^ In this randomised, double-blind study, all the enrolled patients had exon 19 deletions or L858R mutations in their baseline tumour tissue samples. The primary endpoint, investigator-assessed PFS, was significantly longer following osimertinib treatment compared with treatment with first-generation EGFR-TKIs (18.9 versus 10.2 months, HR: 0.46, 95% CI: 0.37–0.57; *P* < 0.0001). No significant difference was reported in terms of ORR (80% versus 76% for osimertinib and standard EGFR-TKI therapy, respectively, odds ratio [OR]: 1.27; 95% CI: 0.85–1.90; *P* = 0.24), but at interim analysis, although only 25% mature, an early trend towards a better OS was shown, with a 37% reduction in the risk of death following treatment with osimertinib versus standard EGFR-TKI therapy (HR: 0.63; 95% CI: 0.45–0.88, *P* = 0.0068). Moreover, osimertinib was better tolerated, with fewer grade 3 or higher adverse effects when compared with first-generation EGFR-TKIs (34% versus 45%).^6^ In view of these results, osimertinib has been propelled into first-line therapy for patients with advanced or metastatic NSCLC with activating *EGFR* mutations, regardless of their T790M status.^13^ Despite these results, the possibility of sequencing treatment with first-/second-generation EGFR-TKIs followed by osimertinib could be a potential alternative with consistent data but applicable only in patients developing T790M mutation.^3^

### Osimertinib efficacy in central nervous system metastases

Central nervous system (CNS) metastases are a common poor prognostic factor in patients with advanced NSCLC with *EGFR* mutations, occurring in approximately 30% of patients during treatment with an EGFR-TKI.^14^ Osimertinib has shown excellent CNS penetration in preclinical studies as well as in clinical trials both as a first- and second-line therapy in patients with *EGFR*-mutant NSCLC.^15,16^ The CNS efficacy of second-line osimertinib was assessed in a subgroup of patients with stable, asymptomatic brain metastases within the Phase 3 AURA3 study.^17^ Median CNS PFS was 11.7 months for osimertinib compared with 5.6 months for chemotherapy (HR: 0.32; 95% CI: 0.15–0.69; *P* = 0.004), and responses were also durable.^17^ At the point of data cut-off (15 April 2016), CNS ORR in patients with one or more measurable CNS lesions was 70 and 31%, in response to treatment with osimertinib and platinum–pemetrexed, respectively (OR: 5.13; 95% CI: 1.44–20.64; *P* = 0.015).^17^

The Phase 3 FLAURA study also revealed the high CNS efficacy of osimertinib in the first-line setting.^18^ In patients with documented CNS metastases, median CNS PFS was 13.9 months (95% CI: 8.3 months to not calculable) with standard EGFR-TKIs but was not reached with osimertinib (95% CI: 16.5 months to not calculable) (HR: 0.48; 95% CI: 0.26–0.86; *P* = 0.014). Remarkably, fewer patients in the osimertinib arm developed new brain lesions compared with the control arm (12% versus 30%), supporting the protective role of osimertinib in the development of new CNS lesions.^18^ Osimertinib also demonstrated an encouraging activity in leptomeningeal metastases (LM), both in first-line^18^ and in second-line^19^ settings. Taken together, these findings further corroborate the use of osimertinib for the front-line treatment of patients with *EGFR*-mutated NSCLC.

## Resistance to osimertinib

Despite the success of osimertinib both in the first-line treatment setting and as a salvage therapy in the presence of the T790M secondary mutation, acquired resistance inevitably occurs—similar to patients treated with first- or second-generation EGFR-TKIs—thus limiting a prolonged clinical benefit achieved with this compound. Moreover, a fraction of patients can experience intrinsic resistance to osimertinib, as observed following treatment with other EGFR-TKIs.

The molecular heterogeneity of NSCLC substantially influences the possible mechanisms of resistance to osimertinib, contributing to the wide spectrum of resistance aberrations discovered so far. In addition, multiple co-existing molecular alterations have been observed in a considerable percentage of patients, both when osimertinib was administered as a front-line therapy as well as after the failure of previous EGFR inhibitors. Hence, repeated tumour biopsies as well as plasma genotyping at the time of progression on osimertinib are crucial steps in unravelling resistance mechanisms and guiding future treatments.

**Fig. 1:**
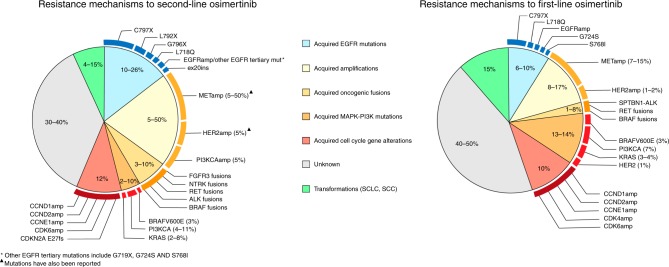
Resistance mechanisms reported for osimertinib according to the line of treatment. The two pie charts depict resistance mechanisms that have been identified in tissue and/or in plasma after resistance to second-line and first-line osimertinib, respectively. Only studies that enrolled more than 15 patients have been taken into account for the ranges of the percentages. In some cases, different molecular aberrations might co-exist in the same patient

**Fig. 2:**
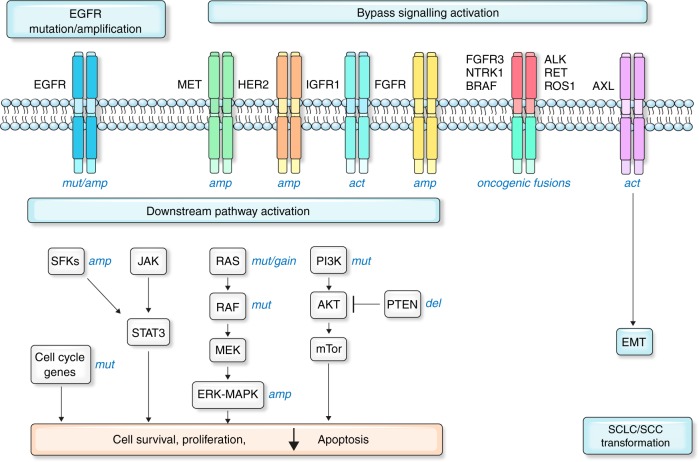
Schematic representation of the known mechanisms of resistance to osimertinib Resistance mechanisms to osimertinib can consist of *EGFR* modifications (mutation/amplification), bypass pathway activation, downstream pathway activation, epithelial-to-mesenchymal transition (EMT), histologic transformation, oncogenic gene fusions and cell-cycle gene aberrations. Abbreviations: act, activation; amp, amplification; del, deletion; mut, mutation

Acquired resistance mechanisms to EGFR-TKIs can be broadly grouped into EGFR-dependent or EGFR-independent mechanisms. Some of the mechanisms are overlapping when osimertinib is administered as a first- or second-line therapy, whereas others have been identified only in one of these settings (Fig. 1). What follows is a description of our current knowledge about the emerging resistance mechanisms to osimertinib in patients with *EGFR*-mutated NSCLC. A schematic representation of the main resistance mechanisms to osimertinib is provided in Fig. 2.

## EGFR-dependent mechanisms of resistance

Insights into EGFR-dependent resistance mechanisms to second-line osimertinib therapy have come from the circulating tumour DNA (ctDNA) genomic profile conducted using plasma from patients with T790M-positive NSCLC enrolled in the AURA3 trial.^20^ In this study, acquired *EGFR* mutations (most commonly C797S; 14%) were observed in 21% of the samples, and all patients with acquired tertiary *EGFR* mutations retained the T790M mutation after progression on osimertinib.^20^ However, 49% of patients lost T790M mutation at the time of progression on osimertinib,^20^ a percentage that was consistent with previous findings.^21–23^ Among the samples that showed a loss of the T790M mutation, ex19del was preferentially present compared with the L858R mutation (83% versus 14%, respectively).^20^ Loss of the T790M mutation manifests as resistance to second-line osimertinib and is usually associated with the emergence of competing resistance mechanisms such as *KRAS* mutations, *MET* amplification, small-cell transformation and gene fusions^20^ (see below). When Oxnard and collaborators^21^ performed next-generation sequencing on tumour biopsy samples after the development of acquired osimertinib resistance in 143 patients who received second-line osimertinib for T790M-positive advanced *EGFR*-mutant NSCLC, they found that the loss of T790M is usually associated with early resistance to osimertinib and a shorter time to treatment discontinuation (6.1 versus 15.2 months).^21^ Further studies confirmed the detrimental impact of T790M loss on patients’ PFS and OS.^20,22,24^ It has also been proposed that the timing of the emergence of osimertinib resistance can shed light on the molecular mechanism, with early resistance usually associated with the loss of the T790M mutation, and late resistance linked to retention of T790M.^21^ In addition, plasma levels of *EGFR* T790M and activating mutations could predict the type of acquired resistance mechanisms.^21^

As expected, when looking at the next-generation sequencing analysis conducted on plasma samples from 91 patients who received front-line osimertinib therapy in the FLAURA trial, no evidence of emergence of the T790M mutation in the osimertinib arm was found. These data were concordant with the pharmacodynamic activity of osimertinib: considering that osimertinib is selective for both *EGFR*-sensitising and T790M mutations, the emergence of T790M under osimertinib treatment was not expected to be a resistance mechanism.^25^

Other EGFR-dependent mechanisms of resistance to osimertinib include the development of *EGFR* tertiary mutations or amplifications,^26,27^ which are more likely to arise in cases in which the *EGFR* T790M mutation is retained.^20,21,23^

### Mutations in C797

The most common tertiary *EGFR* mutation is *EGFR* C797S, which occurs in exon 20 and accounts for 10*–*26% of cases of resistance to second-line osimertinib treatment.^10^ When osimertinib was administered as a front-line treatment, the frequency of the C797S mutation was 7%, making it the second most frequent mechanism, behind *MET* amplification, of drug resistance in this setting.^25^ The *EGFR* C797S mutation, in which cysteine at codon 797 within the ATP-binding site is substituted for by serine, results in the loss of the covalent bond between osimertinib and the mutant EGFR. Predictably, the C797S mutation also confers cross-resistance to other irreversible third-generation TKIs, including rociletinib, olmutinib and narzatinib, by preventing their binding to the EGFR active site.^26,28,29^

**Fig. 3:**
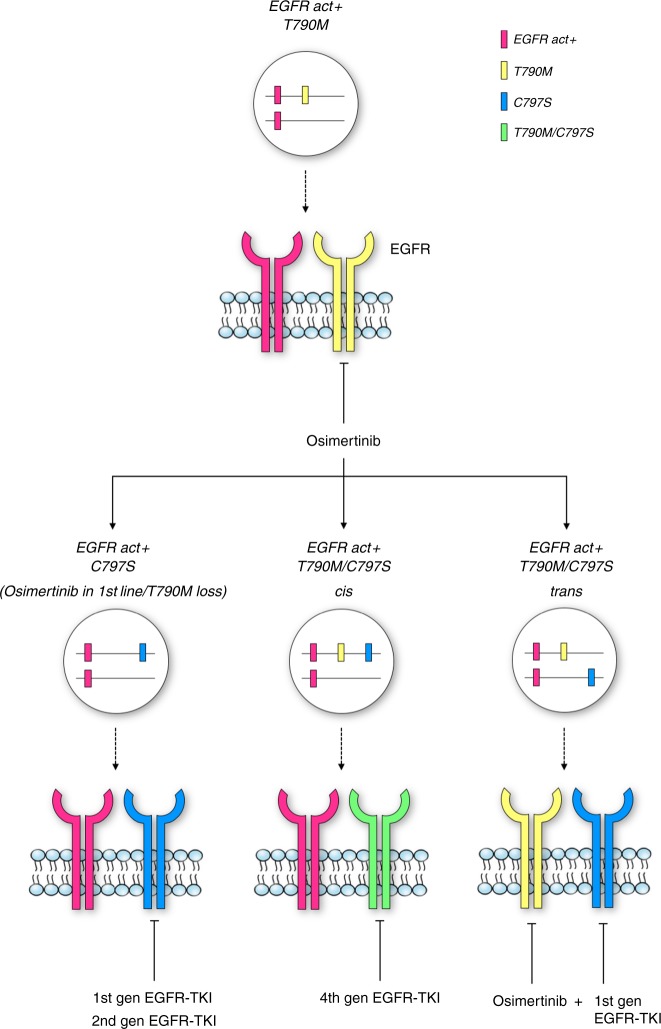
Potential treatment algorithm of T790M *EGFR*-mutated NSCLC. The present figure depicts the most common molecular events within *EGFR* that can occur after the onset of osimertinib resistance. (1) T790M loss/C797S: in this scenario, NSCLC cells re-acquire sensitivity to first-generation and second-generation EGFR-TKIs; (2) T790M/C797S in *cis*: NSCLC cells are amenable to novel fourth-generation EGFR-TKIs which are currently in development and (3) T790M/C797S in *trans*: NSCLC cells become sensitive to combination therapy with osimertinib and first-generation EGFR-TKIs

Importantly, the allelic context in which C797S is acquired has potential implications for treatment (Fig. 3). Given that C797S-positive cells are still sensitive to quinazoline-based EGFR-TKIs, the rare eventuality of the emergence of C797S in *trans* with the T790M mutation allows cells to be targeted with both first-generation and third-generation EGFR-TKIs in order to hit C797S- and T790M-positive alleles, respectively. On the other hand, when the mutations are in *cis*, the cells are found to be resistant to all available EGFR-TKIs alone as well as combined.^28,30,31^ On the basis of preclinical data, clinical efficacy—although limited—of this therapeutic strategy has been reported.^30,31^

Yang and collaborators^26^ investigated the mutation profile of plasma samples obtained from 93 patients with advanced NSCLC treated with osimertinib as a second-line therapy. The authors identified tertiary *EGFR* mutations conferring osimertinib resistance in one-third of the patients. Of these mutations, 24% were the well-known C797S substitution, with co-existing C797G mutation noted in two cases.^26^ The novel tertiary *EGFR* C797G mutation was detected by next-generation sequencing on a pleural biopsy specimen of a single patient who progressed on second-line osimertinib, and it was concomitant with *MYC* and *EGFR* amplifications (see below).^32^

### Mutations in G796

Besides C797X mutations, a number of other rare point mutations in *EGFR* have been identified. On the basis of protein structure prediction, solvent-front mutations in the G796 residue—G796R, G796S and G796D—which is adjacent to C797, have the potential to sterically interfere with the osimertinib–EGFR interaction. G796R has a major impact while G796S has a milder impact on osimertinib–EGFR binding.^26,33,34^ In addition, a G796D mutation has also been detected in a single patient who developed second-line osimertinib resistance.^35^

### Mutations in L792, L718 and G719

The EGFR protein kinase domain has a NH2-terminal lobe (N-lobe) and a larger COOH-terminal lobe (C-lobe) connected by the so-called ‘hinge’ region of the kinase. In silico modelling has demonstrated that mutations in the L792 residue in this region can sterically interfere with a methoxy group on the phenyl ring of osimertinib and disrupt its binding to the kinase domain.^34^ A variety of hinge-pocket mutations in the L792 residue have been reported, among which L792H is the most common.^26^ Interestingly, L792 mutations usually co-exist with other *EGFR* mutations and occur in *cis* with T790M but in *trans* with G796/C797, when present simultaneously in the same patient.^26^ Moreover, L792 mutant variants remained sensitive to gefitinib in vitro.^26^ Mutations in the L718 residue are also responsible for osimertinib resistance in vitro and in vivo.^26,36^ Most of them are L718Q, and the majority of patients with L718 mutations do not have co-existing C797 mutations, suggesting that these mutations could lead independently to osimertinib resistance. The L718 residue is located in the ATP-binding site of the EGFR kinase domain, and in silico modelling has shown that a substitution in this residue can cause spatial restriction and hinder the binding of osimertinib to EGFR. Steric restriction as predicted by in silico modelling might also be the cause of osimertinib resistance in patients with G719A mutations, owing to the close proximity of the G719A residue to the L718 residue.^26,34^ However, in vitro studies have also demonstrated that L718Q might still be sensitive to first- and second-generation EGFR-TKIs, especially when T790M is lost,^37^ and this finding has been confirmed in the clinical setting.^23^ Of note, *EGFR* L718Q has been also found to be a resistance mechanism to first-line osimertinib treatment, accounting for 2% of cases.^25^

### Mutations in G724

G724S mutation in the P-loop of the EGFR kinase domain has been identified after progression to osimertinib in multiple patient cases.^38–42^ Structural analyses demonstrated that G724S mutation induces a conformational change in the receptor which impairs the binding of osimertinib.^39^ Even though the incidence of this rare mutation upon osimertinib treatment is unknown yet, due to the relatively limited evidence available, it is supposed that G724S-mediated resistance preferentially occurs in ex19del but not L858R thus acting in an allele-specific manner.^43^ Interestingly, second-generation EGFR-TKIs retain kinase affinity in G724S mutants, and afatinib was successful in overcoming G724S-mediated resistance to osimertinib in vitro.^43^

### Mutations in exon 20

Besides well-known *EGFR* tertiary mutations ascribed as responsible for osimertinib resistance, other mutations within exon 20 can infrequently occur after progression to osimertinib, but their role in mediating resistance is not established yet.

*EGFR* S768I constitutes a rare mutation in exon 20 that can be found in conjunction with sensitizing *EGFR* mutations at the beginning of EGFR-TKI treatment (occurring in <1% cases).^44^ Even though the exact prognostic and predictive role of S768I is not fully clarified due to its rarity, it has been detected in patients treated with osimertinib in second-line^42^ and also in one patient after progression on front-line osimertinib treatment.^25^

Exon 20 insertion has also been reported in one patient after failure of second-line osimertinib therapy (1%).^20^

### EGFR gene amplification

In the second-line setting, amplification of the wt *EGFR* allele in addition to the presence of the *EGFR-*ex19del allele constituted a novel mechanism of resistance.^45–47^ A study by Kim and colleagues^48^ also revealed increased EGF mRNA expression in tumour tissue samples of patients who progressed on osimertinib.

## EGFR-independent mechanisms of resistance

Osimertinib resistance mediated by EGFR-independent mechanisms can be acquired by activation of alternative bypass pathways, aberrant downstream signalling or histologic transformation. Importantly, these aberrations can co-occur within the same tumour and co-exist with *EGFR* tertiary mutations, underlying the complexity and heterogeneity of cancer evolution in response to EGFR-TKI treatment.

### MET amplification

*MET* gene amplification constitutes the most frequent cause of bypass pathway activation as an acquired resistance mechanism to EGFR-TKIs.^20,49,50^ This event leads to osimertinib resistance by persistent activation of signalling pathways downstream of EGFR, such as those mediated by MAPK, signal transduction and activator of transcription (STAT) and phosphatidylinositol 3-kinase (PI3K)–Akt, independent of EGFR activation and signalling. *MET* amplification can occur with or without loss of the T790M mutation, when osimertinib is given after failure of previous EGFR-TKIs.^23,51–54^ In this clinical setting, the loss of T790M was experienced by 43% of patients, whereas 57% retained the mutation.^20^ In the plasma next-generation sequencing analysis conducted within the AURA3 study, *MET* amplification was observed in nearly 19% of the samples at disease progression and/or treatment discontinuation.^20^ Remarkably, *MET* amplification co-occurred with *EGFR* C797S in 7% of cases,^20^ and is also likely to be associated with *CDK6* and *BRAF* amplifications.^23^ Due to the relative proximity of *CDK6*, *MET* and *BRAF* on chromosome 7q (7q21.2, 7q31.2 and 7q34, respectively), a single genomic event could be hypothesised to be responsible for gene amplifications.^23^ When osimertinib was given as a first-line therapy, *MET* amplification was the most common resistance mechanism, encountered in 15% of patients by next-generation sequence ctDNA analysis.^25^ Moreover, this percentage is expected to be higher in tissue, due to the underestimation of gene amplification in plasma.^25^ *MET* amplification could also represent a potential mechanism of intrinsic resistance to osimertinib.^54,55^

Consistent with these findings, several preclinical studies have demonstrated that the concomitant use of c-Met inhibitors, such as crizotinib, with osimertinib has the potential to overcome resistance in osimertinib-resistant *EGFR-*mutant NSCLC cell lines with *MET* gene amplification.^23,51,54^ Moving to the clinic, the combination of crizotinib and osimertinib could be an effective therapeutic strategy in the eventuality of *MET* amplification onset at the time of acquired resistance to osimertinib.^56–58^ The same drug combination reversed *MET* exon 14 skipping-mediated resistance to osimertinib in *EGFR*-mutated NSCLC cells.^59^ In the clinical setting, *MET* exon 14 skipping has been recently reported in one patient after failure of second-line osimertinib.^42^ Yang et al.^26^ have also identified rare mutations of *MET*—P97Q and I865F—in ctDNA in two cases of second-line osimertinib resistance, but their significance is unknown so far.

### *HER2* amplification

ErbB2 (encoded by *HER2*) is a tyrosine kinase receptor that belongs to the EGFR family and activates the downstream PI3K–Akt and MAPK/extracellular signal-regulated kinase (ERK) kinase (MEK)–ERK/MAPK pathways. *HER2* amplification has been identified in 5% of patients who have acquired resistance to second-line osimertinib,^20^ and it was earlier thought to be mutually exclusive with the T790M mutation, as described for first-generation TKIs.^60,61^ Moreover, *HER2* amplification can co-exist with *EGFR* L792X + C797X + *PIK3CA* amplification (in 1% of cases), *EGFR* G796S + *MET* amplification (1%), and *PIK3CA* amplifications (1%).^20^ Moving to acquired resistance to first-line osimertinib therapy, *HER2* amplification was detected in 2% of cases.^25^ More recently, *HER2* amplification has been reported in one patient who experienced intrinsic resistance to osimertinib.^55^ From the preclinical point of view, Ortiz-Cuaran et al.^54^ studied the impact of *HER2* overexpression on sensitivity to osimertinib and rociletinib in the PC9GR cell line (T790M positive, *EGFR-*mutant) and found that *HER2* overexpression decreased sensitivity to both drugs.

### RAS–MAPK pathway activation

RAS–MAPK pathway aberrations are known to lead to osimertinib resistance in patients with *EGFR-*mutated NSCLC. Eberlain et al.^62^ identified *NRAS* mutations, including the novel E63K mutation, in osimertinib-resistant *EGFR*-mutant NSCLC cell lines together with a gain of copy number of wt *NRAS* or wt *KRAS* in cell populations resistant to gefitinib, afatinib, WZ4002 or osimertinib. In addition, the combination of osimertinib with the MEK inhibitor selumetinib was found to prevent EGFR-TKI resistance both in vitro and in vivo.^62^ Ortiz-Cuaran et al.^54^ identified a *KRAS* G12S mutation on tumour re-biopsy at the onset of acquired resistance to second-line osimertinib. Other *KRAS* mutations, such as G13D, Q61R and Q61K, have also been identified.^21,23,45^ *KRAS* G12D has been reported after osimertinib failure both in first-line and in subsequent lines of therapy,^20,25,47,63^ and pre-existing *KRAS* G12D mutations in association with *PTEN* loss were also detected in two patients who showed primary resistance to second-line osimertinib.^63^

Within the RAS–MAPK pathway, the *BRAF* V600E mutation has been identified as being responsible for osimertinib resistance in 3% of cases,^20,25^ both in first- and in second-line therapy, in association^22,64^ or not^65^ with the *EGFR* T790M mutation when osimertinib was administered after failure of previous EGFR-TKIs. Two cases of concurrent *BRAF* V600E mutation and *MET* amplification as a resistance mechanism to first-line osimertinib therapy have also been reported.^25,66^ Remarkably, cell lines that harboured *BRAF* V600E as a resistance mechanism to osimertinib showed sensitivity to a combination of encorafenib (a BRAF inhibitor) and osimertinib.^64^ Additionally, Kim et al.^48^ have reported *MAPK1* mRNA overexpression in a patient treated with osimertinib in second-line therapy at the time of progression.

### PI3K pathway activation

Bypass activation of the PI3K pathway can occur both via *PIK3CA* mutation/amplification and *PTEN* deletion.^48,67^ Contrary to the mutual exclusivity of most oncogenic driver mutations, the co-occurrence of *PIK3CA* mutations with mutations in other oncogenic driver genes is well described in NSCLC,^68^ in agreement with the supporting role of *PIK3CA* to several oncogenic signalling in NSCLC.^69^ However, although concurrent *PIK3CA* mutations are poor prognostic factors, no significant differences were found in clinical outcomes in patients affected by concurrent *PIK3CA* and *EGFR* mutations treated with EGFR-TKI monotherapy, promoting the use of EGFR-TKIs in such patients.^68^ Among the known *PIK3CA* mutations, E545K, E542K, R88Q, N345K and E418K, which occur at a frequency of 4–11%, have been described to confer resistance to second-line osimertinib therapy.^20,21,23,47,63^ The role of *PIK3CA* E545K in mediating osimertinib resistance has been confirmed in vitro.^23^ Of note, in the next-generation sequencing analysis performed within the AURA3 study, *PIK3CA* amplifications co-occurred with *HER2* amplifications in two out of three cases.^20^ Among patients treated with frontline osimertinib, the *PIK3CA* mutations E453K, E545K and H1047R were identified in six cases, with E545K being the most represented (4%).^25^

### Cell-cycle gene alterations

Alterations in the genes encoding cell-cycle proteins have been found in 12 and 10% of patients who progressed on osimertinib administered as a second-line and first-line therapy, respectively,^20,25^ and have been reported to be associated with poor outcomes following osimertinib treatment.^70^ In particular, several studies have reported amplifications in the genes encoding cyclin D1, cyclin D2, cyclin E1, cyclin-dependent kinase (CDK) 4 and CDK6, and a frameshift deletion in the gene encoding the CDK inhibitor 2A.^20,25^

### Oncogenic fusions

Oncogenic fusions, which are likely to behave as oncogenic drivers, have been identified in 3–10% of cases of acquired resistance to second-line osimertinib, and they can co-occur with *EGFR* C797S, *BRAF* mutation and *MET* amplification.^20^ They include *FGFR3–TACC3* and *RET–ERC1*,^20^ with *CCDC6–RET*, *NTRK1–TPM3*, *NCOA4–RET*, *GOPC-ROS1*, *AGK–BRAF* and *ESYT2–BRAF* also identified as fusions that are potentially responsible for acquired resistance to second-line osimertinib.^21,23,71,72^ The product of the oncogenic fusion *SPTBN1–ALK* has been detected in one patient who received front-line osimertinib but not in patients treated with second-line.^20,25^ Notably, combined EGFR and Ret inhibition with osimertinib and BLU-667 was effective at overcoming resistance due to the presence of the *CCDC6–RET* fusion.^71^ Another study identified receptor tyrosine kinase fusions and BRAF kinase fusions as rare events that mediated resistance to TKIs in patients with advanced NSCLC, including a novel *PLEKHA7–ALK* fusion following osimertinib treatment.^73^ Lastly, an acquired *EML4-ALK* fusion has been confirmed after progression on second-line osimertinib; in this case, the addition of crizotinib to osimertinib led to stability of disease.^74^

### Histologic and phenotypic transformation

Histologic transformation from NSCLC to small-cell lung cancer (SCLC), which arises in 4–15% of cases, is a known mechanism of resistance to first-generation EGFR-TKIs that dramatically impacts patients’ prognosis, and has been reported as an important cause of resistance to osimertinib.^75–79^ A comprehensive understanding of the underlying mechanism(s) responsible for the histologic transformation is still missing. Lee and colleagues^80^ performed whole-genome sequencing of serially acquired biopsy samples from four patients who underwent SCLC transformation under EGFR-TKI treatment, to study the clonal evolution and genetic mechanisms contributing to the event. The authors found complete inactivation of the tumour-suppressor genes *RB1* and *TP53* in the initial NSCLC, as well as at the time of SCLC transformation, pointing to inactivated *RB1* and *TP53* as potential predisposing factors for histologic transformation.^80^ Thus, NSCLC patients who harbour inactivated *RB1* and *TP53* may deserve monitoring for transformation into SCLC during their clinical history.^80,81^ Focusing on SCLC transformation under osimertinib treatment, *RB1* and *TP53* are likely to be inactive at the time of this event, as documented in the case of first-generation and second-generation EGFR-TKIs.^75^ Moreover, the assessment of *RB1* and *TP53* mutational status on ctDNA, as well as the test of neuron-specific enolase (NSE) levels in plasma at the time of progression on osimertinib could be taken into account to unravel a potential SCLC transformation.^75^ Notably, SCLC transformation has been ascribed as a putative mechanism of primary resistance to osimertinib.^75^ On the basis of the limited evidence available to date, in the case of SCLC transformation, a favourable response to combination chemotherapy with platinum and etoposide can be envisioned.^77^ More recently, histologic transformation towards squamous cell carcinoma has been observed after failure of both first-line and second-line osimertinib.^42^

Regarding epithelial-to-mesenchymal transition (EMT), it has been reported that cells from patients with NSCLC with acquired resistance to gefitinib or osimertinib exhibit EMT features, with a decrease in epithelial cell junction proteins such as E-cadherin and an increase in mesenchymal markers such as vimentin, without acquiring any secondary *EGFR* mutations.^82,83^ The EMT features of osimertinib-resistant cells were related to the upregulation of the zinc finger transcription factor Zeb1, and reversing EMT by the dual histone deacetylase (HDAC) and hydroxy-3-methylglutaryl co-enzyme A reductase (HMGR) inhibitor JMF3086 was successful in restoring sensitivity to osimertinib in vitro.^82^ Resistance to osimertinib has also been associated with overexpression of the EMT transcription factor TWIST-1 in *EGFR*-mutated NSCLC cells, and novel strategies to counteract this event using TWIST-1 inhibitors are currently being investigated.^84^

### Other mechanisms

Focal amplification of the *FGFR1* gene accompanied by nearly 20-fold higher levels of fibroblast growth factor 2 (FGF2) mRNA compared with baseline has been reported in an osimertinib-resistant patient.^48^ The co-existence of *FGFR1* amplification and increased levels of FGF2, which is a FGFR1 (FGF receptor 1) ligand, suggests an autocrine loop-mediated mechanism of resistance.^48^ In addition, one case of *FGFR3/FGFR19* amplification has been described.^23^

Src family kinases (SFKs) and focal adhesion kinase (FAK) play a critical role in sustaining the Akt and MAPK pathways under EGFR inhibition in osimertinib-sensitive cells.^83^ The amplification of the SFK member *YES1* has been reported as a resistance mechanism to osimertinib in NSCLC cell lines, and inhibiting SFK or FAK signalling enhanced the effects of osimertinib in vitro.^83^ Another emerging key player involved in osimertinib intrinsic resistance is the receptor tyrosine kinase Anexelekto (AXL).^85^ AXL can interact with other tyrosine kinase receptors, including EGFR and HER3, and sustain survival of tumour cells exposed to osimertinib.^85^ In a recent research study, AXL overexpression was associated with a poor response to osimertinib, whereas combination treatment with an AXL inhibitor and osimertinib prevented the development of intrinsic resistance to osimertinib and the subsequent emergence of drug-tolerant clones in vitro and in vivo.^85^

## Therapeutic strategies to overcome osimertinib resistance

Despite the promising results obtained with osimertinib in advanced patients with NSCLC, resistance ultimately develops due to the treatment selection pressure and the inherent heterogeneity of NSCLC. The intra-patient heterogeneity, and co-occurrence, of multiple resistance mechanisms constitute a major challenge in developing an efficient treatment strategy to counteract tumour progression. Remarkably, the clonal evolution of oncogene-addicted NSCLC can give rise to different molecular aberrations both in space (between primary tumour and metastasis) and in time (after treatment failure), contributing to the complexity of the molecular resistance machinery.^86^

The loss of the T790M mutation during osimertinib therapy is usually associated with treatment failure. Under this circumstance, in the presence of the original driver *EGFR* mutation, it might be feasible to re-treat patients with first-generation EGFR-TKIs.^87^ However, as reported by other authors, the loss of T790M might be accompanied by the emergence of alternative resistance mechanisms such as *MET* amplification and *KRAS* mutation — situations in which first-generation EGFR*-*TKIs are not effective.^21^

Given that *EGFR* C797S is the most common tertiary mutation in patients with T790M-positive osimertinib-resistant NSCLC, overcoming this mutation is the focus of many studies. To this end, fourth-generation EGFR-TKIs have been developed in order to successfully target the C797S mutant EGFR.^88^ Among these, EAI045 is an EGFR allosteric inhibitor which has shown efficacy against C797S-T790M-L858R triple mutant cells when given in combination with the anti-EGFR antibody cetuximab.^89,90^ However, it is not effective against C796S-T790M-ex19del triple mutant cells due to its different structure at the allosteric pocket compared with the C797S-T790M-L858R variant.^89^ JBJ-04-125-02—a novel EGFR allosteric inhibitor—has been recently found to inhibit EGFR C797S-T790M-L858R signalling in vitro and in vivo, and the combination of JBJ-04-125-02 with osimertinib was more effective than either single agent alone.^91^ Brigatinib, a novel dual-target ALK-EGFR inhibitor, has been found to be effective against C797S-T790M-ex19del triple mutant cells in vitro and in vivo.^90,92,93^ Another study also demonstrated that the combination of brigatinib, osimertinib and the vascular endothelial growth factor (VEGF) inhibitor bevacizumab can be effective against lung adenocarcinomas with the triple L858R-T790M-cis C797S *EGFR* mutation.^94^ In the case of uncommon *EGFR* secondary mutations, such as L718Q and L844V, that are present alongside *EGFR*-activating mutations, preclinical studies have demonstrated the effectiveness of afatinib and gefitinib in the absence of the T790M mutation.^87^ Similarly, preclinical studies have shown that the secondary *EGFR* L718V mutation confers resistance to osimertinib but retains sensitivity to the EGFR inhibitor afatinib.^95^

Considering that resistance to osimertinib usually involves multiple mechanisms, such as the activation of alternative cellular pathways or aberrant downstream signalling, osimertinib-based combination therapies are being extensively investigated (Table 1). The combination of osimertinib with pemetrexed or cisplatin has been investigated in *EGFR*-mutant NSCLC preclinical models.^96^ Furthermore, preliminary results have shown that the addition of platinum-based chemotherapy to osimertinib therapy is well tolerated in EGFR-TKI pre-treated patients.^97,98^ A Phase 1 study of carboplatin/etoposide plus osimertinib in *EGFR*-mutated patients with concurrent *RB1* and *TP53* alterations—in order to prevent SCLC transformation—is currently ongoing (NCT03567642). Taking into account the positive OS results derived from the addition of chemotherapy to first-generation TKIs for the first-line treatment of patients with *EGFR*-mutated NSCLC,^99^ randomised trials exploring osimertinib combined with chemotherapy in the same clinical setting are needed.

**Table 1:** Ongoing trials with osimertinib in *EGFR*mut NSCLC

ClinicalTrials.gov ID	Phase	NSCLC study population	Prior EGFR-TKI	Line of treatment	Treatment arm(s)	Description and primary outcome(s)
*Frontline combination strategies*						
NCT03567642	1	*EGFR*mut, concurrent *TP53* and *RB1* alterations	Not allowed	1	Osimertinib + cisplatin/carboplatin + etoposide	Safety of osimertinib combined with platinum-based CT in *EGFR*mut NSCLC patients at increased risk of SCLC transformation - MTD
NCT03122717	1/2	*EGFR*mut	Not allowed	1	Osimertinib + gefitinib	Safety of gefitinib combined with osimertinib - number of patients able to remain on therapy for six cycles
NCT03392246	2	*EGFR*mut, T790M-	Not allowed	1	Osimertinib + selumetinib (MET inhibitor)	Efficacy of selumetinib combined with osimertinib - ORR
*Investigation of novel osimertinib combinations (including the first-line setting)*						
NCT03810807	1	*EGFR*mut	Not allowed	≥1	Osimertinib + dacomitinib	Safety of dacomitinib combined with osimertinib - MTD - ORR
NCT02803203	1/2	*EGFR*mut	Not allowed	≥1	Osimertinib + bevacizumab	Safety of bevacizumab combined with osimertinib - MTD - PFS
NCT02954523	1/2	*EGFR*mut	Not allowed	≥1	Osimertinib + dasatinib	Safety and efficacy of dasatinib combined with osimertinib - MTD - ORR
NCT03455829	1/2	*EGFR*mut	Allowed	≥1	Osimertinib + G1T38 (CDK4/6 inhibitor)	Safety and efficacy of G1T38 combined with osimertinib - DLT - RP2D - AEs - PFS
NCT02971501	2	*EGFR*mut, BM+	Not allowed	≥1	Osimertinib + bevacizumab Osimertinib	Efficacy of bevacizumab combined with osimertinib in patients with BM - PFS
NCT03909334	2	*EGFR*mut	Not allowed	≥1	Osimertinib + ramucirumab Osimertinib	Efficacy of ramucirumab combined with osimertinib - PFS
*Overcoming resistance to osimertinib*						
NCT03891615	1	*EGFR*mut	Mandatory (osimertinib)	≥2	Osimertinib + niraparib (PARP inhibitor)	Safety of niraparib combined with osimertinib after osimertinib resistance - MTD
NCT03944772 (ORCHARD)	2	*EGFR*mut	Mandatory (osimertinib)	2	Osimertinib + savolitinib (MET inhibitor) Osimertinib + gefitinib Osimertinib + necitumumab Durvalumab + carboplatin + pemetrexed	Safety and efficacy of biomarker-matched study treatments after osimertinib resistance - ORR
NCT03532698	2	*EGFR*mut	Mandatory (osimertinib)	2	Osimertinib + aspirin	Efficacy of aspirin combined with osimertinib in overcoming osimertinib resistance - ORR
NCT03778229 (SAVANNAH)	2	*EGFR*mut, *MET*amp/high	Mandatory (osimertinib) Allowed (EGFR-TKI)	2 ≤ *n* ≤ 4	Osimertinib + savolitinib	Efficacy of savolitinib combined with osimertinib in overcoming *MET*amp-mediated resistance to osimertinib - ORR
*Overcoming resistance to prior EGFR-TKI*						
NCT02496663	1	*EGFR*mut	Mandatory	≥2	Osimertinib + necitumumab	Safety and feasibility of necitumumab combined with osimertinib after resistance to prior EGFR-TKI - MTD - AEs
NCT03255083	1	*EGFR*mut, T790M-	Mandatory	≥2	Osimertinib + DS-1205c (AXL inhibitor)	Safety of DS-1205c combined with osimertinib after resistance to prior EGFR-TKI - DLT
NCT02503722	1	- Dose escalation: *EGFR*mut Dose expansion: *EGFR*mut, T790M-	Mandatory	≥2	Osimertinib + sapanisertib (mTOR inhibitor)	Safety and preliminary efficacy of sepanisertinib combined with osimertinib after resistance to prior EGFR-TKI - MTD - DLT
NCT02789345	1	*EGFR*mut, T790M+	Mandatory	≥2	Osimertinib + ramucirumab Osimertinib + necitumumab	Safety and preliminary efficacy of ramucirumab/necitumumab combined with osimertinib after failure of prior EGFR-TKI - DLT
NCT02520778	1	Dose escalation: *EGFR*mut Dose expansion: *EGFR*mut, T790M+	Mandatory	≥2	Osimertinib + navitoclax (Bcl-2 inhibitor)	Safety, tolerability and feasibility of navitoclax combined with osimertinib after failure of prior EGFR-TKI - AEs
NCT03831932	1/2	*EGFR*mut, T790M-	Mandatory	≥2	Osimertinib + glutaminase inhibitor CB-839 hydrochloride	Safety and efficacy of CB-839 hydrochloride combined with osimertinib - RP2D - ORR
NCT03450330 (JACKPOT1)	1/2	*EGFR*mut	Mandatory	≥2	Osimertinib + AZD4205 (JAK inhibitor)	Safety and tolerability of AZD4205 combined with osimertinib after failure of prior EGFR-TKI - AEs
NCT02917993	1/2	*EGFR*mut, T790M+	Mandatory	Ph I: ≥ 2 Ph II: 2	Osimertinib + itacitinib (JAK inhibitor)	Safety and efficacy of itacitinib combined with osimertinib after resistance to prior EGFR-TKI - AEs - DLT - ORR
NCT03784599 (TRAEMOS)	2	*EGFR*mut, *HER2*amp/HER2 overexpr	Mandatory	≥2	Osimertinib + T-DM1	Safety and efficacy of T-DM1 combined with osimertinib in targeting HER2-mediated resistance to prior EGFR-TKI - AEs - ORR
NCT03133546 (BOOSTER)	2	*EGFR*mut, T790M+	Mandatory	≥2	Osimertinib + bevacizumab Osimertinib	Efficacy of bevacizumab combined with osimertinib after progression to prior EGFR-TKI - PFS
NCT03940703	2	*EGFR*mut, *MET*amp	Mandatory	≥2	Osimertinib + tepotinib (MET inhibitor)	Efficacy and safety of tepotinib combined to osimertinib in overcoming resistance to prior EGFR-TKI - DLT - ORR
*Osimertinib monotherapy in special populations*						
NCT03434418	2	*EGFR*mut (G719X, S7681, L861Q)	Not allowed	1	Osimertinib	Activity of osimertinib against uncommon *EGFR* mutations - ORR
NCT03191149	2	*EGFR* ex20 insertion	Allowed	≥1	Osimertinib	Activity of osimertinib against *EGFR* ex20 insertions - ORR

*AEs* adverse events, *BM* brain metastasis, *CT* chemotherapy, *DCR* disease control rate, *DLT* dose-limiting toxicities, *EGFR-TKI* epidermal growth factor receptor—tyrosine kinase inhibitor, *MTD* maximum tolerated dose, *NSCLC* non-small cell lung cancer, *ORR* overall response rate, *OS* overall survival, *PFS* progression-free survival, *RP2D* recommended Phase 2 dose, *SCLC* small-cell lung cancer

*MET* amplification/mutation is a common bypass resistance mechanism to osimertinib, and combining c-Met inhibitors, such as crizotinib, with osimertinib has been found to be effective in osimertinib-resistant *EGFR-*mutated NSCLC patients harbouring *MET* amplification.^56–58^ Clinical benefit from the combination of osimertinib and cabozantinib after simultaneous development of secondary *MET* mutations upon crizotinib treatment has been reported in one patient.^100^ Preclinical evidence has pointed to the combination of the MEK inhibitor selumetinib with osimertinib as a strategy to prevent osimertinib resistance.^62^ Moreover, in a recent paper by Suzawa et al.,^59^ the combination of crizotinib and osimertinib was a successful treatment strategy to tackle acquired *MET* exon 14 skipping that emerged in one *EGFR* L858R + T790M-mutated patient. A combination of osimertinib and trastuzumab-emtansine, a conjugate of the monoclonal antibody trastuzumab (Herceptin) and the cytotoxic agent DM1, was reported to overcome osimertinib resistance in T790M-positive *EGFR*-mutated NSCLC cell lines that gained *HER2* amplification.^101^ Similarly, combining osimertinib with drugs targeting other downstream pathways, such as a BRAF inhibitor^64^ or the AXL inhibitor cabozantinib,^102^ constitutes a promising strategy for overcoming osimertinib resistance, but clinical evidence regarding the effectiveness of these approaches is lacking so far. A number of clinical trials are currently underway combining osimertinib with one or more kinase inhibitors. The TATTON study (NCT02143466) is investigating the combination of osimertinib with savolitinib (c-Met inhibitor) or selumetinib (MEK1/2 inhibitor) in patients who progressed following EGFR targeted treatment. In the same trial, the combination of osimertinib and the immune checkpoint inhibitor durvalumab showed encouraging efficacy results, both in TKI-pre-treated and TKI-naïve patients, but enrolment into this arm has been stopped owing to an increase of pulmonary toxicity. Due to the effect of aspirin in reducing AKT phosphorylation, a combination study of osimertinib with aspirin is also ongoing (NCT03532698). Other trials are exploring osimertinib efficacy in combination with bevacizumab (NCT03133546), the Bcl-2 inhibitor navitoclax (NCT02520778) and the mTorc1/2 inhibitor sapanisertinib (NCT02503722). As resistance mechanisms to osimertinib can also involve dysregulation of cell cycle, the combination of osimertinib with a CDK 4/6 inhibitor is being investigated (NCT03455829).

One of the main limitations of targeted therapy such as TKIs is that the tumour response is not durable with inevitable development of drug resistance. However, it has been recognised that acquisition of drug resistance by cancer cells also makes them more sensitive to alternative drugs, a phenomenon referred to as ‘collateral sensitivity’.^103^ Potentially, this sequential or alternating approach could be used for *EGFR*-mutant NSCLC. After initial treatment with a given TKI, the drug-resistant cells can be eliminated with a second TKI, following which the population should be sensitive to the first TKI again.^103,104^ Other authors have suggested that alternating dosing regimens might be superior to the use of intermittent schedules, by selectively targeting and establishing an equilibrium between both drug-sensitive and drug-resistant cancer cell populations.^105^

## Conclusions and future perspectives

The remarkable success of osimertinib for the treatment of patients with advanced *EGFR*-mutated NSCLC has been mitigated by the development of acquired resistance to this agent. Due to the limited available data about resistance mechanisms to front-line osimertinib, a better molecular characterization of treatment-naïve patients who progressed on osimertinib is eagerly awaited. In the last years, the non-invasive ctDNA genotyping approach has supported tumour re-biopsy in helping to unravel resistance mechanisms to targeted drugs, by providing a wider picture of the heterogeneous molecular events that occur at the time of progression.^106^ Further implementation of liquid biopsy and integration with RNA sequencing data in monitoring the response to osimertinib and detecting the molecular alterations responsible for treatment failure is warranted. For instance, the mutational status of T790M—whose loss is usually associated with early resistance to osimertinib—could be readily monitored in plasma in order to precede a proven radiological progression of disease. Moreover, the implementation of novel technologies such as CRISPR/Cas9 gene editing will help researchers in creating preclinical models for drug testing and will provide additional insights into the complexity of acquired resistance to osimertinib by high-throughput screening of resistant tumours.^107^ CRISPR technology is indeed emerging as a versatile tool to analyse gene function and evaluate new therapeutic strategies. CRISPR/Cas‐mediated gene knockout would be expected to be more efficient than RNA-interference‐mediated gene knockdown. For instance, a recent study combined lentiviral transfection with CRISPR/Cas9 techniques to deliver vectors that encode for Cas9 protein and the specific single-guide RNA (sgRNA) to *EGFR* DNA sequence.^108^ Similarly, the *EGFR*‐mutant genes can be repaired or destroyed with virus‐delivered CRISPR/Cas system.^109^ These ‘molecular surgeries’ on genomic DNA directly target the cause of the resistance in a ‘personalised and possibly permanent manner’. Thus, this novel approach could be combined with traditional chemo- and targeted therapy and should have the potential to significantly improve the survival of patients with *EGFR*‐mutant NSCLC. More detailed data are expected to emerge from the ELIOS study (NCT03239340), a Phase 2, open-label, single-arm study to assess the efficacy, safety and underlying resistance mechanisms to osimertinib when used as first-line therapy in patients with locally advanced or metastatic *EGFR*-mutated NSCLC. In this trial, plasma genotyping together with paired tumour biopsy will be analysed by next-generation sequencing. The recently launched ORCHARD Phase 2 trial (NCT03944772) aims to explore treatment options following disease progression on first-line osimertinib in the same clinical setting by investigating the onset of acquired resistance mechanisms.^110^ In this innovative platform trial, patients will be allocated to a biomarker-matched study treatment—osimertinib plus gefitinib, osimertinib plus savolitinib, osimertinib plus necitumumab, platinum-based doublet plus durvalumab—within each group based on tumour molecular profile.

## Data Availability

Not applicable.
